# Critical Roles and Molecular Mechanisms of Chaperone-Mediated Autophagy in Infections

**DOI:** 10.3390/ijms27031164

**Published:** 2026-01-23

**Authors:** Min Wang, Min Wu

**Affiliations:** 1 Hengyang Medical School, University of South China, Hengyang 421001, China; mm17863810419@163.com; 2 Wenzhou Institute, University of Chinese Academy of Sciences, Wenzhou 325001, China; 3 Tianfu Jincheng Laboratory, Chengdu 610093, China

**Keywords:** chaperone-mediated autophagy, pathogens, infection, lysosomal associated membrane proteins

## Abstract

Chaperone-mediated autophagy (CMA) is a selective lysosomal degradation pathway that relies on the molecular chaperone heat shock cognate 70 kDa protein (HSC70) and the lysosomal receptor LAMP-2A. By recognizing substrate proteins containing KFERQ-like pentapeptide motif, CMA plays a central role in multiple infectious contexts. In host defense and cellular homeostasis, CMA contributes to organelle quality control by selectively degrading damaged or misfolded proteins, including stress- or organelle-associated substrates, thereby limiting pathogen replication while mitigating infection-induced stress and preserving cellular function. Although its detailed mechanisms remain incompletely defined, CMA is thought to involve coordinated steps in which molecular chaperones recognize specific target sequences, recruit autophagy-related components, and deliver substrates for lysosomal translocation and degradation. Recent studies have revealed substantial progress in understanding CMA during viral, bacterial, and fungal infections, identifying key regulatory nodes and signaling pathways. These advances underscore the therapeutic potential of CMA-targeted strategies, such as stabilizing LAMP-2A or enhancing HSC70-mediated substrate recognition. However, the spatiotemporal specificity of CMA’s pro- or antiviral effects remains a major challenge for clinical translation. This review summarizes current progress in this emerging field and highlights unresolved questions, particularly whether tissue- or cell-type-specific regulation of CMA occurs during infection and how precise modulation of CMA activity might achieve optimal anti-infective outcomes.

## 1. Introduction

Infectious diseases remain a major threat to global public health, with millions of deaths worldwide each year. According to the World Health Organization, infectious diseases account for more than 25% of all deaths worldwide [1,2,3]. Among intracellular degradative systems implicated in host defense, chaperone-mediated autophagy (CMA) represents a uniquely selective, vesicle-independent pathway in which cytosolic proteins bearing KFERQ-like pentapeptide motif are recognized by Hsc70 and delivered to lysosomes via the LAMP-2A receptor for unfolding and translocation-dependent degradation [4,5,6].

This receptor-gated, motif-driven selectivity distinguishes CMA from macroautophagy and positions it as a rapid proteostasis “fine-tuner” during stress [7,8]. In infection, however, CMA appears to function as a double-edged sword: it may strengthen host defense by constraining excessive inflammation and stress signaling (e.g., through selective degradation of inflammasome-related components such as NOD-like receptor family pyrin domain containing 3 (NLRP3) and by supporting organelle quality control; and yet it can also be exploited by certain pathogens—particularly viruses—to facilitate replication or immune evasion [9,10,11,12,13,14,15,16]. Accordingly, this review synthesizes recent advances on CMA in bacterial and viral infections, emphasizing mechanistic frameworks and therapeutic/biomarker implications while highlighting a central unresolved gap: the spatiotemporal regulation of CMA during infection and the current lack of robust in vivo tools to quantify CMA flux under physiological infection settings, which together limit causal interpretation and translation [12,17,18,19,20]. Importantly, a central unresolved gap is the spatiotemporal regulation of CMA during infection and the lack of robust in vivo tools to quantify CMA flux in relevant tissues. See Section 5.2.3 for key biological questions that specifically require spatiotemporal resolution.

## 2. Fundamentals and Regulation of Chaperone-Mediated Autophagy (CMA)

### 2.1. Core Mechanisms of CMA

#### 2.1.1. Functional Roles and Synergistic Interactions of Key Participants

The molecular framework of CMA primarily relies on two essential components: the cytosolic chaperone Hsc70 (also known as HSPA8) and the lysosomal receptor LAMP-2A. Cytosolic Hsc70 specifically recognizes substrate proteins carrying a KFERQ-like motif and delivers them to the lysosomal membrane for degradation [21]. In Hsc70-deficient models, substrate recognition and binding efficiency are markedly reduced, whereas reintroduction of wild-type Hsc70 restores CMA activity, underscoring its indispensable role in the early substrate recognition stage of this pathway [22,23].

LAMP-2A is a central receptor component and a major determinant of CMA capacity. Its abundance on the lysosomal membrane strongly influences substrate translocation efficiency and overall CMA flux. Functional studies show that increasing LAMP-2A enhances substrate uptake and degradation, whereas LAMP-2A depletion impairs CMA activity [24,25].

Beyond these core molecules, several auxiliary cofactors contribute to the fine-tuning and stability of CMA. Hsp90 stabilizes the LAMP-2A translocation complex and assists productive translocation; Glial fibrillary acidic protein (GFAP) and EF1α participate in the dynamic assembly and disassembly of LAMP-2A multimers, ensuring efficient channel formation and substrate translocation; and the luminal homolog lys-Hsc70 “pulls” unfolded substrates across the lysosomal membrane to complete translocation [26,27,28].

CMA activity is regulated by multiple signaling pathways, among which the phosphoinositide 3-kinase (PI3K)/protein kinase B (PKB; AKT)/mTOR cascade is thought to negatively regulate CMA flux by downregulating LAMP-2A and Hsc70 expression [29]. At the systems level, stable-isotope tracing and live-cell imaging studies have revealed that efficient coordination between Hsc70 and LAMP-2A enables timely clearance of misfolded proteins, thereby sustaining amino acid recycling, redox balance, and global proteostasis. Conversely, inhibition of CMA leads to aggregate accumulation, mitochondrial dysfunction, and reduced resistance to oxidative stress-effects that are particularly pronounced under infectious or inflammatory conditions [30,31].

Moreover, growing evidence indicates that CMA and macroautophagy operate in a complementary rather than redundant manner. When macroautophagy is impaired, CMA is frequently upregulated to preserve protein quality control and cellular homeostasis, although this compensatory relationship varies among cell types and stress contexts [32]. Mechanistically, both reviews and experimental studies have proposed that mTORC2, calcineurin, and transcription factors such as FoxO1 act as central signaling hubs mediating the switch between autophagic pathways during metabolic stress or pathogen invasion [21,33].

Collectively, Hsc70 and LAMP-2A form the structural and functional backbone of CMA, while auxiliary proteins and interconnected signaling networks ensure its efficiency and precision. Through this coordinated system, CMA emerges as a vital regulatory mechanism that enables cells to adapt to stress and maintain immune homeostasis.

#### 2.1.2. Substrate Selectivity and Degradation Mechanisms of CMA

A defining feature of CMA is its remarkable substrate selectivity. Cytosolic proteins containing the canonical KFERQ or KFERQ-like motif are specifically recognized by Hsc70, which forms a substrate-chaperone complex. This complex is directionally transported along microtubules toward the lysosomal membrane with the assistance of molecular motors such as dynein and kinesin. Pharmacological disruption of the cytoskeletal network markedly reduces substrate trafficking efficiency, highlighting the indispensable role of the cytoskeleton in CMA-mediated substrate delivery [8,28]. Moreover, dynein-associated cofactors-such as Cytoplasmic dynein 1 light intermediate chain 2(DYNC1LI2)-have been identified as key regulators of LAMP-2A transport and receptor localization on the lysosomal surface [34].

At the lysosomal membrane, the substrate-Hsc70 complex initially binds to monomeric LAMP-2A. Under conditions of nutrient deprivation or oxidative stress, LAMP-2A undergoes controlled oligomerization, forming multimeric translocation complexes capable of mediating substrate import [35]. Multiple studies have demonstrated that this translocation process is adenosine triphosphate (ATP)-dependent and relies on luminal Hsc70 (lys-Hsc70) to pull unfolded substrates across the lysosomal membrane in a stepwise, energy-driven manner [8,28].

CMA substrate selectivity is further modulated by post-translational modifications (PTMs). Modifications such as acetylation, oxidation, and phosphorylation can alter substrate affinity for Hsc70 or affect the efficiency of translocation across the lysosomal membrane [5,36].

Once inside the lysosomal lumen, ATP hydrolysis and luminal Hsc70 activity facilitate complete substrate unfolding and translocation, followed by proteolytic degradation into small molecular products by cathepsins D and L [37]. These degradation products are then recycled to the cytosol via lysosomal transporters such as solute carrier family 38 member 9 (SLC38A9), integrating back into amino acid metabolism and biosynthetic pathways [38].

To prevent metabolic imbalance caused by CMA overactivation, cells employ negative feedback mechanisms, including downregulation of LAMP-2A abundance or polymerization capacity through mTORC2 and p38 mitogen-activated protein kinase (MAPK) signaling pathways [6]. In addition, competitive inhibition among substrates has been reported: abundant, slow-turnover proteins may monopolize CMA machinery, thereby restricting the degradation of critical regulatory factors [26].

CMA activity gradually declines with aging, leading to the accumulation of damaged or misfolded proteins and increased cellular vulnerability to pathological conditions such as infection and neurodegenerative diseases [39].

#### 2.1.3. Comparison with Canonical Autophagy Pathways

Compared to canonical macroautophagy, CMA exhibits distinct molecular and kinetic characteristics, particularly in substrate selectivity and degradation dynamics. Classical macroautophagy relies on the formation of double-membraned autophagosomes that engulf portions of the cytoplasm for bulk delivery to lysosomes, whereas CMA degrades individual cytosolic proteins via a receptor-gated, chaperone-assisted lysosomal translocation mechanism (see Section 2.1.1) [5,40]. Focusing on CMA, we only touch base on macroautophagy features relevant to CMA crosstalk; detailed macroautophagy mechanisms are not discussed.

Evidence indicates that distinct proteolytic systems contribute differentially to cellular protein turnover. Under nutrient deprivation or stress, macroautophagy becomes strongly upregulated and takes on a major role in protein degradation, whereas CMA is essential for selective removal of specific cytosolic substrates [41]. Macroautophagy proceeds through a multi-step process involving the unc-51 like autophagy activating kinase 1 (ULK1) complex, the class III PI3K complex, and the ATG12-ATG5-ATG16L1 conjugation system, which cooperatively regulate phagophore initiation, membrane elongation, autophagosome closure, and fusion with lysosomes. This is a dynamic and sequential pathway that typically operates on a timescale of several hours to complete cargo degradation [42]. By contrast, CMA bypasses vesicle formation and directly translocates substrate proteins across the lysosomal membrane. This enables rapid and highly selective clearance of misfolded or damaged proteins, as demonstrated by live-cell CMA reporter assays that monitor substrate delivery and lysosomal association in real time [32].

At the functional level, macroautophagy is indispensable for global metabolic remodeling and energy homeostasis, particularly under conditions of starvation or hypoxia [43]. CMA, on the other hand, provides fine-tuned proteostasis control by selectively degrading dysfunctional proteins and preventing toxic aggregate accumulation, thereby supporting both basal homeostasis and adaptive stress responses [6,44]. This regulatory precision is especially critical in long-lived, post-mitotic cells such as neurons and hepatocytes, which cannot dilute damaged proteins through cell division [28].

Extensive crosstalk and compensatory mechanisms exist between the two autophagic systems. When CMA activity declines, macroautophagy is often upregulated to sustain cellular homeostasis, and vice versa [45,46]. Under certain stress conditions, such as oxidative stress or aging, cells may preferentially activate CMA, suggesting an environment-dependent hierarchy in the utilization of distinct autophagic pathways [47]. Mechanistically, mTORC2 and transcription factor EB (TFEB) have been identified as central signaling hubs that coordinate the balance and division of labor between CMA and macroautophagy [48,49,50]. Additionally, post-translational modifications (PTMs) of CMA substrates further refine degradation selectivity-for instance, mild oxidative modification enhances Hsc70 binding, whereas excessive oxidation impedes substrate translocation [51,52].

Accumulating evidence indicates that CMA activity declines progressively with age, a phenomenon tightly linked to the pathogenesis of various chronic disorders. In particular, defective CMA contributes to the impaired clearance of aggregated proteins and consequent loss of cellular function in neurodegenerative diseases [53]. In such contexts, cells often rely on compensatory degradation systems, including the ubiquitin-proteasome and macroautophagy pathways; however, these mechanisms cannot fully substitute for the precise regulatory capacity of CMA [25,45].

#### 2.1.4. Measuring CMA Activity and Flux: Experimental Readouts and Specificity Controls

CMA activity should be evaluated using flux-oriented assays rather than steady-state levels of LAMP-2A or Hsc70 alone, because changes in these proteins may also reflect general lysosomal stress or broader proteostasis remodeling [21,33]. A widely used approach is the KFERQ-tagged fluorescent reporter system (e.g., photoconvertible KFERQ-like–Dendra2 reporter (KFERQ–Dendra2)), which enables time-resolved quantification of CMA-dependent lysosomal delivery by monitoring reporter puncta formation and lysosomal colocalization (e.g., with LAMP-1/LAMP-2A–positive compartments) under stress conditions [32,54]. Complementarily, ex vivo lysosomal uptake and degradation assays using isolated lysosomes provide a direct readout of CMA substrate translocation, as import and degradation require an intact LAMP-2A-dependent translocation machinery and the Hsc70/lys-Hsc70 system [6]. To distinguish CMA from macroautophagy, specificity controls are essential: genetic disruption of CMA components (LAMP-2A depletion or Hsc70 interference) should abolish reporter translocation or lysosomal uptake [32,35,54], whereas inhibition of macroautophagy initiation (e.g., *autophagy related 5* (*ATG5*)/*autophagy related 7* (*ATG7*) deficiency) blocks macroautophagy flux [42], but is not expected to eliminate CMA-specific reporter translocation, which should remain dependent on LAMP-2A/Hsc70 integrity [32,54]. Conversely, lysosomal inhibitors (e.g., bafilomycin A1) confirm lysosome dependence but do not by themselves discriminate CMA from other lysosomal degradation routes [32,54,55]. Collectively, these approaches define a practical framework for assessing CMA flux, while also highlighting the need for quantitative, tissue-resolved in vivo CMA reporters in infection models.

#### 2.1.5. Criteria for Assigning CMA-Specific Effects: Evidence Hierarchy and Exclusion Rules

In infection and immunology studies, changes in autophagy–lysosome pathways are frequently inferred from global pharmacological perturbations or steady-state markers. However, these approaches often cannot completely distinguish CMA from macroautophagy, microautophagy, or general lysosomal stress. To reduce over-attribution and to ensure mechanistic specificity, we applied an explicit evidence hierarchy when interpreting the literature and when using CMA-causal language throughout this review.

Evidence hierarchy (from highest to lowest confidence):

Tier 1—CMA-specific causal evidence (high confidence). Studies that demonstrate (i) a CMA flux–oriented readout (e.g., KFERQ-reporter trafficking/turnover, lysosomal uptake assays of CMA substrates) and (ii) genetic CMA perturbation with dependency/rescue (e.g., LAMP-2A knockout/knockdown with rescue; functional Hsc70 perturbation) supporting that the observed phenotype is LAMP-2A/Hsc70-dependent. Only Tier 1 evidence is interpreted as “CMA-dependent” or “CMA-specific” in this review.

Tier 2—Strong CMA association with partial specificity (moderate confidence). Studies that include either (i) a flux-oriented CMA readout without genetic dependency tests, or (ii) genetic LAMP-2A/Hsc70 perturbation but rely mainly on indirect/steady-state markers for CMA activity. These are interpreted as “CMA-associated” and “consistent with CMA involvement,” but not definitive.

Tier 3—Component-level changes (low confidence). Studies that report changes in LAMP-2A/Hsc70 abundance or lysosomal markers without a CMA flux measurement and without genetic dependency. Because LAMP-2A/Hsc70 are broadly stress-responsive and can change under diverse proteostatic or inflammatory conditions, Tier 3 findings are described as “alterations in CMA-related components” rather than “CMA activation/inhibition.”

Tier 4—Indirect/autophagy–lysosome perturbation (hypothesis-generating). Studies that infer CMA involvement from non-specific pharmacological agents (e.g., lysosomotropic agents, inhibitors/activators that broadly impact autophagy or lysosomal function) or from macroautophagy readouts (e.g., microtubule-associated protein 1 light chain 3 (LC3)/sequestosome 1 (SQSTM1; p62) changes) without CMA flux assays and genetic dependency tests. These are considered supportive of a role for the autophagy–lysosome axis but are not assigned as CMA-specific mechanisms.

Exclusion rules and wording constraints used in this review:

(1) CMA-exclusive claims (e.g., “CMA mediates/regulates/controls X”) are used only when Tier 1 criteria are met.
(2) When evidence is Tier 2–4, we use conservative phrasing such as “CMA may contribute,” “CMA has been implicated,” “CMA-associated changes,” or “lysosome-dependent degradation.”
(3) Steady-state increases/decreases in LAMP-2A or Hsc70 alone are not interpreted as CMA flux changes. We explicitly distinguish CMA capacity/components from CMA activity/flux.
(4) Pharmacological manipulation of autophagy/lysosomes is not attributed to CMA unless CMA dependency is demonstrated through LAMP-2A/Hsc70 genetic tests and/or CMA flux readouts.

Using these criteria, each pathogen- or pathway-specific claim in the main text is framed according to its highest supporting evidence tier, and key studies are categorized accordingly in Table 1 to transparently separate CMA-specific mechanisms from broader autophagy–lysosome phenomena.

### 2.2. Cellular Signaling Pathways Regulating CMA

#### 2.2.1. Regulation by the mTOR Signaling Pathway

As a central sensor of cellular nutrient and energy status, the mechanistic target of rapamycin (mTOR) complex plays a pivotal role in autophagy regulation. Under nutrient-rich conditions, mTOR complex 1 (mTORC1) is activated, resulting in high-level phosphorylation of its downstream effectors, including 4E-BP1, ribosomal protein S6 kinase (S6K), and unc-51 like autophagy activating kinase 1 (ULK1), thereby suppressing the initiation of autophagy [58]. In particular, phosphorylation of ULK1 at Ser757 prevents its interaction with AMP-activated protein kinase (AMPK), constituting a classical mechanism of macroautophagy inhibition [59]. Although direct evidence linking CMA to ULK1 regulation remains limited, these findings suggest that elevated mTORC1 activity may broadly constrain CMA function [60].

**Figure 1:**
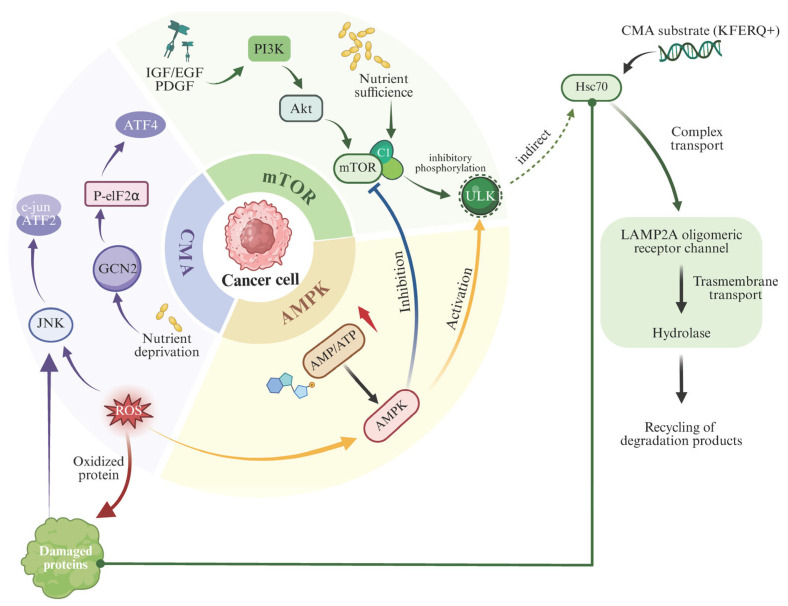
Integrated regulation of CMA. The diagram shows that the CMA core is composed of Hsc70 and LAMP-2A. PI3K-Akt-mTOR inhibits, while AMPK and the stress axis promote activation. The substrate enters the lysosome via LAMP-2A for degradation and recycling, demonstrating the nodal crossover with macroautophagy and the overall homeostasis regulation. Created in BioRender. Li, C. (2026) https://BioRender.com/9k8bizc (accessed on 15 January 2026).

However, it should be noted that ULK1 is not a canonical component of CMA, be cause CMA does not require ULK1-driven autophagosome biogenesis and instead proceeds via LAMP-2A–dependent substrate translocation at the lysosomal membrane. Nevertheless, emerging evidence supports bidirectional crosstalk between ULK1-centered nutrient/stress signaling and CMA-related proteostasis control. For example, ULK1 itself has been reported to undergo CMA-dependent turnover in a context- and modification-dependent manner, suggesting that CMA may tune ULK1 abundance rather than being directly “activated” by ULK1 [61]. In addition, ULK1 has been linked to broader chaperone/proteostasis circuitry under cellular stress [62], implying potential indirect coupling between ULK1-associated pathways and CMA capacity. Accordingly, ULK1 is best presented as a putative crosstalk node rather than a definitive upstream switch for CMA, and direct mechanistic evidence for ULK1-driven CMA activation is limited. For clarity, an overview of major signaling pathways and regulatory inputs that shape CMA activity is summarized in Figure 1.

Unlike macroautophagy-which relies on ULK1-driven formation of autophagosomal membranes-CMA operates primarily at the lysosomal membrane via receptor-mediated translocation through LAMP-2A. Thus, mTOR regulation of CMA is largely exerted at the level of LAMP-2A stability and trafficking. Both mTORC1 and mTORC2 have been shown to exert multi-targeted, hierarchical control over CMA, reflecting the pathway’s layered complexity [63].

Broader network analyses reveal extensive crosstalk between the mTOR pathway and PI3K-Akt, AMPK, and TSC1/2 signaling cascades. Activation of PI3K-Akt promotes mTORC1 and consequently suppresses CMA, whereas AMPK activated under energy deficient conditions inhibits mTORC1 and thereby indirectly enhances CMA [48]. The TSC1/2 complex, a negative regulator of mTORC1, integrates multiple stress signals, including oxidative stress, hypoxia, and energy depletion, to fine-tune this balance [64].

During infection, mTOR signaling has been implicated in the regulation of CMA. In Salmonella-infected macrophages, mTORC1 inhibition (e.g., rapamycin) increases lysosomal degradation capacity and enhances removal of bacterial effector proteins [65]. While the study did not directly evaluate CMA flux, these observations indicate that mTOR inhibition may create a permissive metabolic state that supports CMA-mediated host defense. Conversely, chronic inflammation has been associated with sustained mTORC1 activation and impaired CMA function, leading to the accumulation of protein aggregates and mitochondrial dysfunction [66,67].

Pharmacological studies further demonstrate that mTOR inhibitors including rapamycin, Torin1, a potent inhibitor of both mTORC1 and mTORC2 and AZD8055, a selective ATP-competitive mTOR kinase inhibitor not only hold therapeutic value in cancer and immunosuppression but also show potential in restoring CMA activity during aging, infection, and neurodegenerative diseases [68,69]. However, given the pleiotropic roles of mTOR in cell growth, metabolism, and immune signaling, precise control of its activity remains a major challenge. Therefore, considering the key inhibitory role of the mTORC2-Akt-PH domain and leucine-rich repeat protein phosphatase 1 (PHLPP1) axis in CMA regulation, future investigations are needed to define its molecular control mechanisms and assess whether targeting this pathway could be a viable strategy to reactivate CMA [60].

#### 2.2.2. Regulation of CMA by the AMPK Signaling Pathway

As a central sensor of cellular energy stress, AMP-activated protein kinase (AMPK) is activated in response to glucose deprivation, hypoxia, oxidative stress, or mitochondrial dysfunction, primarily through phosphorylation at Thr1 [25,70]. Once activated, AMPK orchestrates autophagy regulation through multiple mechanisms. In macroautophagy, AMPK directly phosphorylates ULK1 at Ser317 and Ser777, while simultaneously inhibiting mTORC1, thereby relieving its suppressive modification of ULK1 and promoting autophagy initiation [59]. Although CMA does not depend on ULK1-driven autophagosome formation, AMPK activation under similar energy stress conditions has likewise been shown to enhance CMA activity.

Using GFP-KFERQ reporter systems and lysosomal colocalization assays, AMPK activation induced by pharmacological agents such as 5-aminoimidazole-4-carboxamide ribonucleotide (AICAR) or metformin-significantly increases CMA substrate translocation and degradation, indicating a positive regulatory role of AMPK in CMA [71]. Further evidence suggests that this effect is mediated not only through mTOR inhibition but also through direct modulation of LAMP-2A stability and lysosomal membrane recruitment [4]. Experimental findings demonstrate that AMPK activation is accompanied by increased LAMP-2A levels and enhanced recruitment of Hsc70 to the lysosomal membrane, suggesting a role for AMPK in the post-translational regulation of CMA components [23]. Conversely, silencing PRKAA1 (encoding AMPKα1) or expression of dominant-negative AMPK markedly reduces starvation-induced CMA activity, confirming the causal role of AMPK in this pathway.

Beyond direct regulation, AMPK also modulates CMA indirectly through transcriptional control. Studies have shown that AMPK activation promotes dephosphorylation and nuclear translocation of transcription factor EB (TFEB) and transcription factor E3 (TFE3), thereby enhancing transcription of lysosomal and autophagy-related genes [72,73]. In parallel, forkhead box O3 (FOXO3), a direct substrate of AMPK, undergoes AMPK-dependent phosphorylation that increases its transcriptional activity [74]. Together, these findings outline an upstream regulatory cascade-AMPK → TFEB/TFE3 and FOXO3-that coordinates autophagy-related gene expression. However, direct transcriptional regulation of CMA core components (LAMP-2A and Hspa8) by TFEB or FOXO3 remains to be conclusively demonstrated, warranting further ChIP-seq and functional validation studies.

At the metabolic level, AMPK-mediated regulation of CMA promotes energy mobilization and metabolic adaptation. Under energy stress, AMPK induces phosphorylation of perilipin 2 (PLIN2), enabling its recognition by Hsc70 and subsequent degradation through CMA, which facilitates lipid droplet mobilization and maintains cellular energy balance [75]. This mechanism exemplifies the specialized role of CMA in metabolic homeostasis.

From both pathological and therapeutic perspectives, the AMPK-CMA axis holds significant potential across diverse disease contexts. In neurodegenerative diseases, such as Parkinson’s and Alzheimer’s disease, AMPK activation has been reported to partially restore CMA activity, promote clearance of pathogenic protein aggregates, and improve cellular function [76]. Pharmacological AMPK activators-including resveratrol, berberine, and AICAR-have demonstrated CMA enhancing effects in both cellular and animal models. Moreover, in infection models, AMPK activation has been proposed as a host-directed strategy to facilitate CMA-dependent pathogen clearance, although this concept remains under active investigation [77,78].

#### 2.2.3. The Role of the PI3K-Akt Signaling Pathway in Regulation of CMA

Recent studies have begun to elucidate the complex relationship between the phosphoinositide 3-kinase (PI3K)-Akt signaling pathway and CMA. Upon extracellular stimulation by insulin, growth factors, or cytokines, PI3K becomes activated and catalyzes the conversion of phosphatidylinositol 4,5-bisphosphate (PIP2) to phosphatidylinositol 3,4,5-trisphosphate (PIP3), which recruits Akt (protein kinase B) to the plasma membrane. Akt is subsequently phosphorylated at Thr308 by 3-phosphoinositide-dependent protein kinase 1 (PDK1) and at Ser473 by mTORC2, leading to its full activation [79,80].

In autophagy research, Akt activation is generally recognized as inhibitory. In macroautophagy, Akt suppresses autophagy initiation by enhancing mTORC1 activity, phosphorylating ULK1, and directly targeting Beclin 1 (BECN1) [81,82]. Evidence regarding its role in CMA, however, remains emerging. Several studies have reported that enhanced Akt signaling-such as that induced by IGF-1 or epidermal growth factor (EGF)-is accompanied by decreased LAMP-2A levels and reduced CMA substrate degradation, whereas pharmacological inhibition of PI3K/Akt restores LAMP-2A expression and increases CMA flux [83,84]. These findings suggest that activation of the PI3K-Akt pathway may function as a negative regulator of CMA.

Notably, extensive crosstalk exists between the PI3K-Akt axis and other metabolic and stress-related pathways, including AMPK and MAPK, collectively determining autophagic activity under varying cellular conditions [85]. In metabolic disease models, excessive Akt activation often coincides with autophagic defects, oxidative protein accumulation, and chronic inflammation. Although direct evidence for CMA impairment in these contexts remains limited, Akt-mediated CMA inhibition has been proposed as a plausible underlying mechanism [86,87].

In the context of infection, certain pathogens appear to exploit PI3K-Akt signaling to evade CMA-dependent clearance. For instance, *Mycobacterium tuberculosis* and hepatitis C virus (HCV) have been shown to enhance host Akt activity, thereby suppressing lysosome-dependent degradation pathways and potentially impairing CMA [12].

Overall, current evidence supports a suppressive role of the PI3K-Akt pathway in CMA regulation, though the precise molecular mechanisms remain incompletely defined. Future research integrating CMA-specific reporter systems and pathogen infection models will be essential to dissect how this pathway governs LAMP-2A stability and substrate translocation dynamics [21].

### 2.3. Influence of Extracellular Environmental Factors on CMA Activity

#### 2.3.1. Nutrient Deprivation as a Potent Inducer of CMA

Nutrient deprivation represents one of the most powerful extracellular stimuli that enhance CMA activity [54,88]. When cells experience shortages of essential nutrients-such as amino acids, glucose, or lipids-they rapidly initiate adaptive intracellular responses aimed at conserving energy, maintaining homeostasis, and ensuring survival. A key component of this adaptive process is the activation of stress-responsive kinases and the subsequent upregulation of autophagic pathways, particularly CMA, to alleviate metabolic deficits.

Under amino acid starvation, cells detect the accumulation of uncharged tRNAs via activation of general control nonderepressible 2 (GCN2), which triggers the integrated stress response (ISR). This pathway mediates eIF2α phosphorylation and activates downstream transcription factors activating transcription factor 4 (ATF4) and C/EBP homologous protein (CHOP), which are well-established regulators of macroautophagy and amino acid metabolism [89]. Although current evidence suggests that this stress axis may indirectly influence the transcription of CMA-related genes such as LAMP-2A and Hspa8, direct binding or causal validation remains lacking. Therefore, while a potential link between ISR and CMA can be hypothesized, its precise molecular mechanism requires further elucidation using approaches such as ChIP-seq and transcriptional profiling.

Recent studies have shown that lipid deprivation, particularly cholesterol depletion, induces the activation of transcription factors TFEB and TFE3, which in turn upregulate genes involved in lysosomal biogenesis [50,90]. Although most work has focused on macroautophagy, emerging evidence suggests that TFEB may also contribute indirectly to CMA regulation by stabilizing and promoting the expression of lysosomal components [91]. These findings imply a broader role for CMA in lipid metabolic adaptation, especially in the coordination of cholesterol sensing and lysosomal homeostasis. While direct mechanistic data remain limited, this process may be intimately linked to the pathogenesis of metabolic disorders such as nonalcoholic fatty liver disease (NAFLD) and atherosclerosis [92].

From a systems-level perspective, nutrient scarcity activates multiple metabolic sensing pathways, including GCN2-eIF2α, AMPK, and mTOR, which together form a hierarchical network that coordinates autophagic regulation [93]. Within this framework, CMA exerts a selective proteolytic role-preferentially degrading nonessential or damaged proteins while preserving critical regulatory molecules-to sustain amino acid recycling and metabolic homeostasis [94]. These mechanisms underscore CMA as an essential cellular strategy for survival and metabolic adaptation under adverse conditions.

#### 2.3.2. Impact of CMA by Oxidative Stress

Oxidative stress is one of the most critical exogenous stimuli regulating CMA. When cells are exposed to ultraviolet radiation, heavy metals, chemical toxins, or proinflammatory cytokines, excessive reactive oxygen species (ROS) are generated, surpassing homeostatic thresholds and causing oxidative damage to deoxyribonucleic acid (DNA), proteins, and lipids. Such perturbations disrupt cellular integrity and metabolic equilibrium. Under these conditions, multiple studies have reported a selective upregulation of CMA, which facilitates the removal of oxidatively damaged proteins and preserves proteostasis [71,95]. Notably, compared with macroautophagy, CMA demonstrates superior substrate specificity for oxidized proteins, positioning it as a key defensive mechanism under oxidative stress. However, the precise temporal dynamics and molecular mechanisms of CMA activation during oxidative stress remain incompletely characterized, underscoring the need for systematic experimental investigation.

Several studies have documented that autophagy-related protein 8 (Atg8/ATG8)/LC3 proteins undergo diverse post-translational modifications (post-translational modifications (PTMs))-including phosphorylation, acetylation, and ubiquitination-that affect their lipidation and membrane-binding capacity. Yet, direct evidence that such PTMs substantially weaken LC3-membrane affinity and suppress canonical macroautophagy remains lacking [96]. Consequently, the hypothesis that “PTM-mediated suppression of macroautophagy permits relative preservation of CMA” remains largely theoretical, although recent reviews have proposed this mechanism as a potential explanation for stress-induced switching between degradation pathways [97].

CMA activation depends on the oligomerization of LAMP-2A at the lysosomal membrane, a structural rearrangement essential for substrate translocation [98]. Under oxidative stress, both LAMP-2A and Hsc70 expression are upregulated, enhancing the removal of oxidatively modified proteins and providing adaptive cytoprotective effects [95]. Importantly, CMA responses appear cell type-dependent: in neurons, CMA is often robustly activated, whereas in hepatocytes, chronic ROS exposure may impair lysosomal stability and lead to CMA dysfunction [71].

Mechanistically, the redox-sensitive transcription factor nuclear factor erythroid 2–related factor 2 (NRF2) has been experimentally validated as a positive regulator of CMA through transcriptional upregulation of Lamp2A, forming a feed-forward regulatory loop that sustains CMA flux [99]. In contrast, the potential involvement of other transcription factors-such as HIF-1α-in CMA regulation remains speculative, with direct mechanistic evidence still lacking.

Collectively, these findings highlight CMA as a critical defense system for detoxifying oxidized proteins and maintaining redox homeostasis. By selectively degrading damaged biomolecules, CMA mitigates oxidative stress induced cell death, senescence, and inflammation. Consequently, modulation of CMA represents a promising therapeutic strategy for disorders characterized by oxidative injury, including neurodegenerative diseases, cardiovascular pathologies, and infection-related tissue damage [100].

#### 2.3.3. Integrated Regulation of CMA by Extracellular Stimuli

Beyond nutrient deprivation and oxidative stress, a variety of extracellular cues-including growth factor availability, cytokine stimulation, temperature fluctuations, osmotic stress, and mechanical tension-can individually or cooperatively modulate CMA. Through these mechanisms, cells achieve environment-specific control of proteostasis in response to microenvironmental dynamics.

At the level of growth factors, attenuation of insulin/IGF-1 signaling has been experimentally demonstrated to enhance CMA activity and is associated with proteomic remodeling in longevity models [26]. Conversely, several studies and reviews have proposed that high-level stimulation by EGF or IGF-1 may downregulate LAMP-2A through the PI3K-Akt-mTOR axis, thereby restricting CMA flux-a finding that suggests a bidirectional mode of growth factor-dependent regulation [22,26].

Regarding inflammatory cytokines, Interferon gamma (IFN-γ) and Tumor necrosis factor alpha (TNF-α) act synergistically in mesenchymal stem cells to induce LAMP-2A cleavage and degradation via Akt activation, leading to inhibition of CMA function [84]. In contrast, certain stress-related stimuli can enhance CMA activity by promoting LAMP-2A oligomerization or upregulating its transcription, reflecting the conditional adaptability of CMA regulation under distinct inflammatory or stress contexts.

At the level of intracellular stress signaling, endoplasmic reticulum (ER) stress has been shown to recruit p38 MAPK to the lysosomal membrane, where it directly phosphorylates LAMP-2A at Thr211 and Thr213, thereby increasing its membrane stability and oligomerization efficiency, ultimately enhancing CMA activity [101]. This finding provides molecular evidence that stress-activated kinases can directly regulate CMA.

Although direct evidence linking mechanical cues (such as matrix stiffness or shear stress) to CMA is still lacking, existing studies have shown that cells sense and respond to their physical microenvironment through integrin-mediated adhesion structures, which activate focal adhesion kinase (FAK) and integrin-linked kinase (ILK) signaling cascades and influence autophagy-particularly macroautophagy [102]. Indeed, overall autophagic flux has been shown to depend on extracellular matrix (ECM) stiffness, giving rise to the emerging concept of “mechanoautophagy”, which describes the crosstalk between mechanical forces and autophagic regulation [103]. While these findings primarily concern macroautophagy, they offer conceptual insight into how CMA might likewise respond to mechanical microenvironmental cues. Supporting this notion, certain studies describe that mechanical stretching triggers chaperone-assisted selective autophagy (CASA) to degrade damaged cytoskeletal proteins, suggesting a potential role for chaperone-mediated degradation under mechanical stress-though whether this process involves canonical CMA remains to be determined [104].

These diverse extracellular inputs often converge through signaling crosstalk. For example, combined effects of oxidative stress and inflammatory cytokines may synergistically enhance CMA via activation of forkhead box O (FOXO) transcription factors, while hyperosmotic stress has been reported to increase autophagic flux through polycystin-2 (PC2)-mediated Ca^2+^ signaling that suppresses mTOR activity [105]. Although this evidence primarily concerns macroautophagy, it raises the possibility that osmotic stress could indirectly offset the inhibitory influence of growth factor signaling on CMA. Such regulatory paradigms carry significant implications for disease biology. In neurodegenerative disorders, such as Parkinson’s disease and Alzheimer’s disease, persistent inflammatory signaling and growth factor dysregulation may impair CMA, leading to the accumulation of toxic protein aggregates and neuronal dysfunction. Emerging microenvironmental intervention strategies, including anti-cytokine therapies, growth factor mimetics, and biomaterials with tunable mechanical properties, may help restore CMA homeostasis and alleviate proteotoxic stress in disease-specific contexts. Collectively, these nutrient- and stress-sensing pathways provide a mechanistic framework for interpreting how pathogens reshape CMA during infection, as summarized in Figure 1.

## 3. Roles of CMA in Different Types of Infection

To facilitate cross-comparison while minimizing over-attribution from non-specific au tophagy/lysosome perturbations, Table 1 summarizes infection content supported by Tier 1–3 evidence, including the reported net directionality (pro- or anti-viral; or context-dependent/unclear) and the key signaling pathways/nodes implicated, together with the corresponding citations. Cases that primarily reflect macroautophagy or general lysosomal maturation/acidification (not CMA-specific) are curated in Supplementary Table S1, whereas hypothesis-level links between infection-associated stress programs and CMA regulation are summarized in Supplementary Table S2.

### 3.1. The Role of CMA in Bacterial Infections

#### 3.1.1. Experimental Evidence and the “Dual Recognition” Model

An increasing body of research has proposed a “dual recognition model” in which, during infection, pattern recognition receptors (PRRs) not only detect pathogen-associated molecular patterns (PAMPs) but also sense damage-associated molecular patterns (DAMPs) released from the host. This dual sensing triggers immune and stress responses such as endoplasmic reticulum (ER) stress, the unfolded protein response (UPR), and reactive oxygen species (ROS) production [106]. These stress signals may indirectly enhance host surveillance of protein misfolding, suggesting that CMA could be activated under certain infectious conditions-although direct experimental evidence for this remains lacking.

In the *Mycobacterium tuberculosis* (*Mtb*) infection model, immunity-related GTPase family M (IRGM) has been shown to modulate mitochondrial function and autophagic flux to enhance antibacterial defense [107]. While this study did not specifically address CMA, it highlights the protective contribution of autophagy in Mtb infection and provides a conceptual basis for investigating CMA’s potential involvement. Similarly, in *Escherichia coli* infection models, a dual cellular response has been observed: Toll-like receptor 4 (TLR4) engagement by lipopolysaccharide (LPS) rapidly initiates immune signaling, while molecular chaperones are recruited to refold or degrade damaged host proteins [108,109]. These findings indicate that cells can simultaneously perceive extracellular pathogen stimuli and intracellular proteotoxic stress [110]; however, whether CMA is directly involved in this integrated response remains to be determined through systematic investigation.

To date, no experimental studies have definitively demonstrated that silencing LAMP-2A or disrupting Toll-like receptor 2 (TLR2)/TLR4 signaling directly impairs CMA activity; nonetheless, several lines of evidence suggest indirect links. Activation of TLR4 (e.g., by LPS stimulation) enhances macrophage antibacterial capacity and general autophagic activity [111,112], whereas LAMP-2A deficiency leads to protein homeostasis disruption and elevated inflammatory signaling [13]. These observations imply that CMA plays a vital role in maintaining intracellular equilibrium. It can therefore be hypothesized that, in CMA-deficient models, bacterial clearance would be compromised and misfolded proteins would accumulate-a direction warranting future experimental validation.

Mechanistically, CMA and inflammatory signaling are likely functionally coupled. By degrading misfolded or aberrant signaling molecules, CMA may limit excessive or prolonged inflammatory activation, thereby maintaining homeostatic balance [13]. Furthermore, the expression of key CMA components, LAMP-2A and HSPA8, is modulated by oxidative stress and metabolic perturbation [6], underscoring the pathway’s sensitivity to the cellular stress landscape.

Certain bacterial virulence factors, such as ROS-generating enzymes and pore-forming toxins, can disrupt host protein conformation or induce oxidative modifications, potentially enhancing their affinity for heat shock cognate 70 kDa protein (HSC70) and thereby increasing the likelihood of CMA engagement. In aged hepatic models, CMA has been shown to selectively eliminate oxidatively damaged proteins, thereby maintaining proteostasis [6,26]. Compared to the relatively nonselective nature of macroautophagy, CMA demonstrates rapid and substrate-specific responsiveness during the early stages of cellular stress by recognizing proteins bearing KFERQ-like motifs.

**Figure 2:**
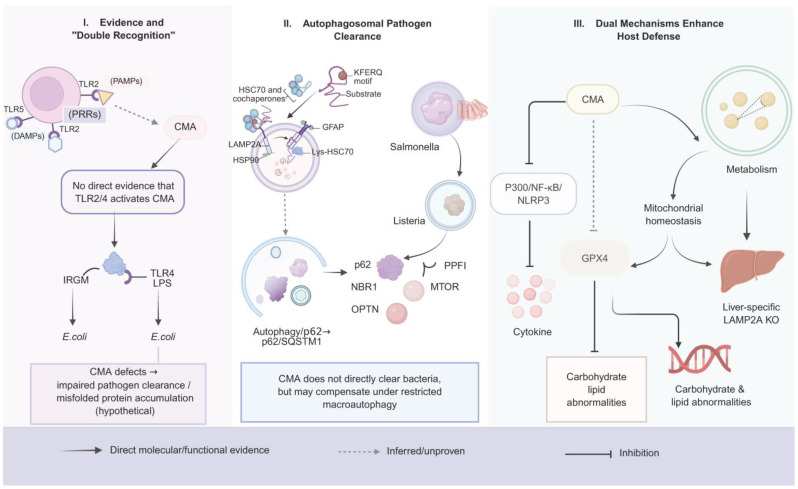
Proposed model of CMA involvement in bacterial infection and host defense. CMA functions in bacterial infection through a three-stage process of “recognition-clearance-regulation”: (**I**). There is no direct evidence that TLR2/4 directly activates CMA; (**II**). CMA compensatorily clears oxidized or abnormal proteins when autophagy is restricted; (**III**). It maintains mitochondrial and metabolic homeostasis and inhibits excessive inflammation through molecules such as GPX. Created in BioRender. Li, C. (2026) https://BioRender.com/6bo5ucx (accessed on 15 January 2026).

Collectively, these findings suggest that CMA may function as a quality control mechanism that protects host cells against pathogen-induced stress, contributing to both proteostatic defense and anti-inflammatory regulation. This dual functionality positions CMA as a potential therapeutic target for immunomodulatory and anti-infective interventions in the future. This proposed ‘dual recognition’ framework and the current evidence gaps regarding PRR–CMA linkage are summarized in Figure 2.

#### 3.1.2. Experimental Evidence of Autophagic Clearance of Pathogens

Autophagosomes play a central role in innate immune defense by recognizing, sequestering, and delivering intracellular pathogens to lysosomes for degradation. Live fluorescence imaging and colocalization analyses showed that in *Salmonella* infection models, bacteria became associated with LC3-positive structures at early post-infection time points (≈1 h), and genetic deletion of Atg5 markedly increased intracellular bacterial proliferation, indicating an autophagy-dependent restriction mechanism [113]. However, certain *Salmonella* strains have evolved strategies to evade this process: bacterial effectors such as SopB, SseF, and SseG suppress autophagy initiation, thereby facilitating immune escape [114,115].

During *Listeria monocytogenes* infection, host defense relies on Galectin-8 and nuclear dot protein 52 kDa (NDP52) to detect ruptured phagosomal membranes, initiating xenophagy that re-sequesters and degrades the bacteria, effectively limiting their replication [116]. Further studies have revealed that selective autophagy receptors-including p62, neighbor of BRCA1 gene 1 (NBR1), and optineurin (OPTN)-recognize ubiquitinated or oxidatively modified bacterial proteins, thereby promoting their targeted degradation [117]. Although there is currently no direct evidence that CMA can recognize or clear bacteria, previous findings indicate that CMA efficiently degrades aberrant or oxidized proteins under stress conditions. This suggests that CMA may provide compensatory proteostatic support when macroautophagy is impaired or proteotoxic stress becomes excessive [4].

Real-time CMA reporter systems based on KFERQ-Dendra and KFERQ-PS-CFP2 have demonstrated that CMA is rapidly engaged upon cellular stress, with substrates translocating to LAMP-2A positive lysosomes, allowing dynamic visualization of CMA activation [32,54]. This rapid responsiveness offers indirect evidence that CMA may participate in early-stage cytoprotection during infection.

Moreover, *Salmonella* can activate the FAK-Akt-mTOR signaling pathway through its SPI-2-encoded effectors, leading to suppression of autophagy. In contrast, macrophages deficient in FAK exhibit enhanced autophagy and reduced bacterial burden, underscoring the complexity of host signaling networks governing autophagic responses [118].

Taken together, the autophagy lysosome system constitutes a fundamental mechanism for clearing intracellular pathogens. The rapid activation and selective recognition features of CMA suggest that it may serve as a complementary mechanism to macroautophagy under stress conditions. Although direct experimental evidence of CMA targeting bacterial pathogens is currently lacking, its established role in protein quality control offers new perspectives on infection-associated stress responses and positions CMA as a promising candidate target for future therapeutic intervention [21,118].

#### 3.1.3. Dual Mechanisms Cooperatively Enhancing Host Defense

Recent studies have revealed that CMA plays a critical regulatory role in PRR (pattern recognition receptor)-induced inflammatory signaling. In vitro models have demonstrated that stimulation with TLR4 ligands-such as lipopolysaccharide (LPS)-leads to exacerbated activation of the p300/NF-κB/NLRP3 signaling axis when CMA activity is reduced, resulting in heightened inflammatory responses. Conversely, enhancement of CMA suppresses NLRP3 inflammasome activation, thereby decreasing the release of proinflammatory cytokines such as TNF-α and IL-6 [13,119,120]. These findings position CMA as a key modulator that fine-tunes innate immune signaling intensity and prevents hyperinflammatory damage.

Beyond immunoregulation, CMA contributes to the maintenance of cellular metabolism and mitochondrial homeostasis. Impaired CMA leads to metabolic imbalance, reduced ATP production, and compromised mitochondrial integrity, whereas sustained CMA activity supports energy recovery and overall cellular function [7,25]. Moreover, CMA selectively degrades damaged or oxidatively modified proteins, preventing their accumulation and preserving stem cell viability and systemic proteostasis [17]. In contrast, CMA dysfunction triggers metabolic dysregulation, elevated stress responses, and exacerbated inflammation [7].

A mechanistic link has also been established between CMA and lipid peroxidation. Critically, CMA-mediated degradation of glutathione peroxidase 4 (GPX4) correlates with dynamic changes in 4-HNE levels, suggesting that CMA may participate in the adaptive regulation of cellular oxidative stress responses [106]. In vivo, liver-specific LAMP-2A knockout mice exhibit pronounced carbohydrate and lipid metabolic abnormalities, further underscoring CMA’s indispensable role in maintaining host energy balance [7].

Collectively, these findings support a model in which PRR-mediated pathogen sensing and CMA-driven intracellular quality control operate in a complementary and interdependent manner to maintain immune equilibrium and metabolic stability. This cooperative mechanism not only shapes inflammatory outcomes but also integrates proteostatic and metabolic homeostasis, offering critical insight into host–pathogen interactions and highlighting CMA modulation as a promising therapeutic avenue for restoring immune-metabolic balance. Taken together, these lines of evidence support an integrated model in which PRR-driven pathogen sensing, macroautophagy-mediated pathogen clearance, and CMA-dependent proteostatic/metabolic control cooperate to shape host defense (Figure 2).

### 3.2. CMA in Ribonucleic Acid (RNA) Virus Infections

#### 3.2.1. Potential Functions and Emerging Evidence of CMA in RNA Virus Infection

While macroautophagy–ribonucleic acid (RNA) virus interactions have been extensively reviewed, the roles of CMA in RNA virus infection remain far less defined; therefore, we focus here on CMA-specific evidence and testable hypotheses. In contrast to macroautophagy, the involvement of CMA in RNA virus infection remains in its early stages of exploration. Experimental evidence from LPS-stimulated microglial inflammation models indicates that activation of CMA suppresses the NF-κB/NLRP3 signaling axis, thereby attenuating proinflammatory cytokine production [120]. Although not a viral model, this finding implies that CMA may play a broader immunomodulatory role in infection-associated inflammation.

Recent work on SARS-CoV-2 has provided additional clues: LAMP-2A was found to interact with the viral 5′/3′ untranslated region (UTR) RNA protein complexes, and overexpression of LAMP-2A reduced viral RNA abundance [56]. These observations suggest that SARS-CoV-2 may modulate LAMP-2A dependent CMA as a strategy to influence host proteostasis and viral replication.

Beyond coronaviruses, evidence from other RNA viruses also supports a possible CMA connection. In hepatitis C virus (HCV) infection, the viral nonstructural protein 5A (NS5A) protein interacts directly with host Hsc70 and promotes the lysosomal degradation of HNF-1α through a LAMP-2A dependent mechanism, thereby altering host proteostasis and metabolic regulation [15,121]. This provides molecular-level evidence for direct CMA participation during HCV infection. In contrast, while influenza A virus (IAV) infection has been shown to extensively reprogram host transcriptional and metabolic networks [122,123,124], there is currently no direct evidence that LAMP-2A or CMA contributes to these processes. The potential link between IAV and CMA therefore remains speculative and warrants further investigation.

#### 3.2.2. Mechanistic Hypothesis: Does CMA Recognize Viral Proteins?

To date, no experimental evidence demonstrates that RNA viral proteins contain KFERQ-like motifs or are directly recognized as CMA substrates. However, CMA may indirectly influence viral replication by degrading host proteins modified by virus-induced stress, such as oxidized or misfolded proteins. This hypothesis could be tested through viral proteomic screening, co-immunoprecipitation assays, and fluorescent tracking systems to determine whether viral infection triggers CMA-dependent proteome remodeling.

#### 3.2.3. CMA Modulation as an Antiviral Strategy

Given CMA’s roles in proteostasis maintenance and inflammatory regulation, pharmacological modulation of CMA-through specific activators or inhibitors-offers a promising avenue for antiviral intervention. Unlike direct antiviral agents that target viral genomes or proteins, host-directed CMA modulation may provide a broader and more mutation-resistant therapeutic approach, particularly valuable in the face of rapid viral evolution and drug resistance. Targeting CMA thus represents a novel conceptual framework for developing host-centered antiviral strategies that harness intrinsic proteolytic pathways to suppress infection and restore cellular homeostasis.

### 3.3. The Role of CMA in Antifungal and Antiparasitic Immunity

#### 3.3.1. Potential Roles of CMA in Antifungal Infection

Direct experimental evidence for the role of CMA in fungal infection remains scarce. However, studies have shown that fungal pathogens such as *Candida albicans* and *Cryptococcus neoformans* can activate Dectin-1-dependent recognition of β-glucans, leading to the production of reactive oxygen species (ROS) and induction of endoplasmic-reticulum (ER) stress in host cells [125]. Both ROS and ER stress have been reported to upregulate key CMA components-including Hsc70 and LAMP-2A-and to promote their membrane oligomerization, thereby enhancing CMA activity [101]. Although direct evidence that fungal infection activates CMA is lacking, these stress-responsive pathways likely serve as potential upstream signals for CMA induction.

In other pathogen or stress models, CMA has been shown to selectively degrade aggregated or damaged host proteins, maintaining proteostasis and reducing cytotoxicity [4,30,126]. It is therefore reasonable to infer that, during fungal infection, CMA activation could support antifungal immunity indirectly by removing stress-induced host proteins, sustaining lysosomal flux, and preserving cellular function. Whether CMA can directly target fungal molecules or virulence factors remains unproven and warrants further investigation.

Based on the established application of the KFERQ-Dendra2 reporter system and LAMP2A/HSC70-deficient models in CMA research [4,7,25,127], future studies could integrate these tools into fungal infection settings to investigate whether and how CMA contributes to antifungal immunity.

#### 3.3.2. Potential Roles of CMA in Antiparasitic Infection

Parasitic infections also trigger host-cell stress responses, which may indirectly modulate CMA activity. In *Plasmodium berghei* liver-stage infection, hepatocytes undergo ER stress and activate the unfolded protein response (UPR), characterized by the upregulation of XBP1s and the liver-specific UPR transcription factor **cAMP-responsive element-binding protein H (CREBH)**, reflecting the engagement of adaptive stress pathways to counter protein-folding pressure [128]. These same signals have been shown in other models to regulate LAMP-2A and Hsc70 expression [22], suggesting a possible link between UPR activation and CMA enhancement during infection.

Accumulating evidence indicates that lysosome-dependent degradation pathways and selective autophagy processes, including macroautophagy and CMA, can modulate antigen processing and MHC I/II presentation, thereby shaping T-cell-mediated immune responses [129,130]. While CMA’s direct participation in these processes has not been experimentally confirmed, it has been reported to regulate MHC II trafficking, internalization, and stability [131]. This provides a theoretical basis for CMA’s potential role in modulating antiparasitic immune responses.

#### 3.3.3. Potential Involvement of CMA in *Toxoplasma gondii* Infection

*Toxoplasma gondii* infection induces oxidative stress and activates multiple stress-signaling pathways. For instance, phosphorylation of eIF2α is markedly increased during early infection [132]. In addition, the parasite effector dense granule protein 24 (GRA24) sustains host p38 MAPK activation, whereas dense granule protein 15 (GRA15) triggers NF-κB signaling [133,134]. These stress pathways are known to modulate autophagy; notably, the eIF2α/ATF4 axis has been shown to upregulate autophagy-related genes in other models [135]. Thus, *T. gondii*-induced stress responses may indirectly promote CMA activity to support protein quality control and cellular homeostasis.

At present, there is no direct evidence that CMA exerts parasite-specific clearance functions in toxoplasmosis. Future studies using CMA reporter systems and LAMP2A/Hsc70-deficient models will be essential to determine the precise contribution of CMA during parasitic infection.

#### 3.3.4. Therapeutic Prospects for Host-Directed Modulation

Although direct experimental validation of CMA’s role in fungal and parasitic infections is still lacking, its functions in stress adaptation, lysosomal flux maintenance, and antigen presentation position it as a promising host-directed therapeutic target. Small-molecule CMA activators such as atypical retinoid 7 (AR7) and its derivatives have already shown efficacy in restoring proteostasis in neurodegenerative and metabolic disease models [136,137]. Evaluating these compounds in fungal or parasitic infection models could clarify their therapeutic potential and safety. In particular, CMA modulation may serve as an adjunctive strategy for infection control in cases of antimicrobial resistance or immunocompromise, offering a novel approach to harnessing the host’s intrinsic proteolytic machinery for pathogen restriction.

## 4. Interplay Between CMA and Host Immune Responses During Infection

### 4.1. Regulation of Immune Cell Activity and Function by CMA

#### 4.1.1. Potential Roles with Macrophages

Macrophages, as central components of the innate immune system, play pivotal roles in pathogen clearance, inflammatory regulation, and antigen presentation. Emerging evidence indicates that inflammatory stimuli or cellular stress can modulate the expression and activity of core CMA machinery components (including LAMP-2A and HSC70), thereby shaping macrophage metabolic homeostasis and inflammatory signaling programs [13,120].

In several non-infectious stress models, including nutrient deprivation and oxidative challenge, both in vitro and in vivo studies have demonstrated a rapid and dynamic enhancement of CMA activity, as visualized using KFERQ-Dendra reporter systems. These systems reveal an increase in colocalization between KFERQ substrates and lysosomal markers (e.g., LAMP-1), accompanied by a marked rise in fluorescent puncta within several hours to tens of hours following stimulation [54]. These findings indicate that CMA can respond swiftly to stress stimuli; however, whether it is activated in a similar temporal and spatial manner during infection remains to be experimentally confirmed.

In antigen presentation, macroautophagy plays a well-established role in facilitating MHC class II loading and CD4^+^ T cell activation [138]. Moreover, CMA has been shown to promote the delivery of cytosolic antigens to MHC class II molecules [131]. However, whether CMA operates similarly during pathogen infection remains to be fully elucidated.

**Figure 3:**
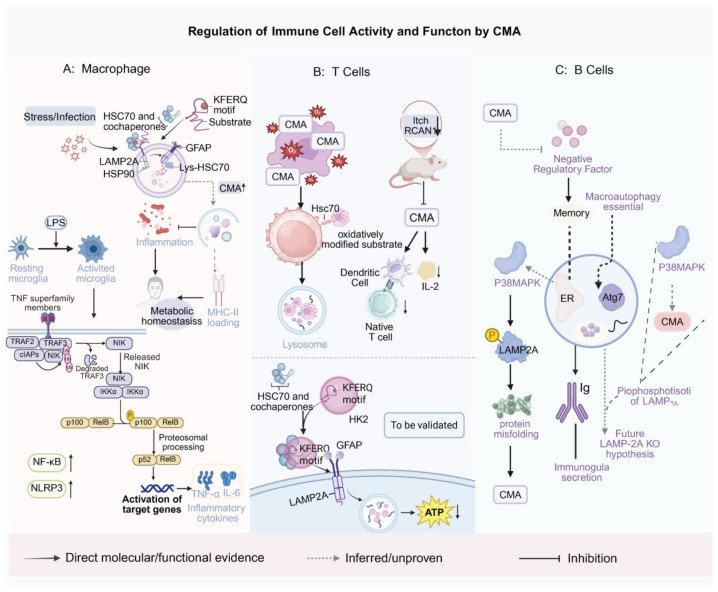
CMA-dependent regulation of macrophage, T-cell, and B-cell function. The diagram illustrates how CMA regulates inflammation, signal persistence, and metabolic homeostasis in macrophages, T cells, and B cells, respectively. The B cell mechanism is largely speculative and requires further validation using a LAMP-2A conditional knockout model. Created in BioRender. Li, C. (2026) https://BioRender.com/358ruw1 (accessed on 15 January 2026).

Furthermore, CMA activity appears to be closely linked to the inflammatory polarization of phagocytes. In LPS-stimulated microglial models, CMA deficiency enhances NF-κB/NLRP3 signaling, resulting in robust secretion of proinflammatory cytokines such as TNF-α and IL-6 [120]. Similarly, in macrophage-associated atherosclerosis models, LAMP-2A deficiency leads to heightened inflammation and elevated cytokine release [13]. These findings collectively underscore CMA’s role as a negative regulator of excessive inflammation, essential for maintaining immune and metabolic homeostasis in macrophages. For clarity, the proposed roles of CMA in regulating macrophage inflammation/antigen presentation, T-cell signaling and metabolism, and B-cell ER proteostasis and memory are summarized in Figure 3.

#### 4.1.2. T Cell Metabolism and Signaling Regulation

T cell activation and differentiation rely on profound metabolic reprogramming and tightly regulated signal transduction. Emerging evidence indicates that CMA contributes to sustaining T cell activation by selectively degrading negative regulators of T cell receptor (TCR) signaling, including Itch and RCANIn vitro experiments have shown that silencing LAMP-2A or Hsc70, which impairs CMA function, leads to attenuated TCR downstream signaling, reduced IL-2 secretion, and diminished T cell proliferation. Conversely, when CMA remains intact, T cells maintain prolonged activation and enhanced effector function [52].

Beyond direct modulation of signaling pathways, CMA also supports T cell function under stressful microenvironments. In oxidative stress models, CMA activity is rapidly upregulated; Hsc70 recognizes oxidatively modified substrates and delivers them to lysosomes for degradation, thereby preventing abnormal protein aggregation within the cytoplasm [30]. Further, selective inhibition of CMA causes the accumulation of damaged proteins and decreased cell survival under oxidative stress, whereas preservation of CMA integrity mitigates oxidative injury and enhances cell viability [25]. These findings suggest that CMA not only regulates immune signaling by removing inhibitory factors but also preserves proteostatic balance to protect signaling fidelity under stress conditions.

CMA has also been implicated in the regulation of cellular metabolism. Previous studies demonstrated that CMA targets Hexokinase 2 (HK2), a key enzyme in glycolysis containing a canonical KFERQ-like motif, for Hsc70-dependent lysosomal degradation via LAMP-2A. Upon CMA activation, HK2 degradation leads to a reduction in glycolytic flux and intracellular ATP levels, ultimately triggering energy stress and metabolic imbalance. Conversely, CMA inhibition-such as through LAMP-2A knockdown-stabilizes HK2, partially restores metabolic activity, and delays cell death [139]. Although this mechanism has not yet been validated in T cells, it points to a potential role for CMA in immune cell metabolic regulation.

It should be noted, however, that no direct evidence currently supports CMA-mediated modulation of extracellular signal-regulated kinase (ERK) phosphorylation or other canonical TCR downstream signaling cascades. Thus, the precise contribution of CMA to T cell proliferation and effector differentiation remains to be clarified. Taken together, although CMA has been shown to sustain TCR signaling by promoting the degradation of Itch and regulator of calcineurin 1 (RCAN1) and thereby enhancing T-cell activation and cytokine production [52], direct evidence that CMA modulates downstream ERK phosphorylation or other canonical TCR signaling nodes remains lacking. CMA may instead support T-cell responses under stress by maintaining proteostasis and metabolic balance [140].

#### 4.1.3. The Potential Role of CMA in B Cell Responses and Immune Memory

During activation and differentiation, B cells undergo profound metabolic and proteostatic stress-particularly at the plasma cell stage, where the massive synthesis of immunoglobulins (Igs) imposes a heavy burden on the endoplasmic reticulum (ER) protein-folding machinery. Previous studies have established that macroautophagy is essential for B cell function and humoral immune responses. For instance, deletion of the autophagy-related gene Atg7 does not impair B cell development but markedly compromises plasma cell formation and long-term antibody responses, underscoring the indispensable role of autophagy in B cell survival and sustained immune competence [141]. Similarly, other studies have demonstrated that macroautophagy contributes to the maintenance of immunological memory, further emphasizing its supportive role in humoral immunity [142].

In contrast, the involvement of CMA in B cell biology remains largely unexplored. Mechanistic studies in other contexts have shown that ER stress has been shown to activate CMA via p38 MAPK-mediated phosphorylation of LAMP2A at T211/T213, which promotes lysosomal membrane assembly of LAMP2A and facilitates removal of misfolded proteins [101]. Given that B cell activation is accompanied by intense Ig synthesis and ER expansion, it is plausible that CMA acts as a complementary quality-control mechanism, helping to preserve ER homeostasis during the antibody production process. However, no direct experimental evidence has yet confirmed that CMA enhances antibody secretion or mitigates ER stress in activated B cells; thus, this hypothesis remains theoretical at present [143].

During long-term B cell responses and immune memory formation, macroautophagy has been clearly shown to be indispensable, yet it remains uncertain whether CMA provides a similar auxiliary function. Current speculation suggests that CMA may indirectly support the establishment and persistence of memory B cells by degrading inhibitory regulators of differentiation or survival, but this idea lacks functional validation to date [131].

Future investigations employing B cell-specific LAMP-2A knockout models and in vivo immune-response tracking could elucidate the contribution of CMA to antibody secretion, ER quality control, and memory maintenance. Such studies would advance our understanding of CMA as a potential host-directed immunoregulatory mechanism, offering new conceptual and therapeutic avenues for enhancing humoral immunity. Altogether, these observations suggest that CMA shapes immune responses through cell type–specific effects on inflammatory signaling, proteostasis, and metabolism, though substantial gaps remain—particularly in B cells—requiring targeted in vivo models and spatiotemporal assays (Figure 3).

### 4.2. Dynamic Regulation of CMA by Host Immune Responses

#### 4.2.1. Proinflammatory Cytokines and Their Potential Links to CMA Components

Proinflammatory cytokines, beyond orchestrating immune cell activation and effector functions, may also indirectly modulate CMA by influencing protein quality control pathways. Emerging evidence indicates that key CMA components undergo dynamic regulation under stress and inflammatory conditions. For example, the membrane abundance and stability of LAMP-2A fluctuate in response to stress signaling, leading to corresponding alterations in CMA flux [144]. Concurrently, Hsc70 expression and its recruitment to lysosomal membranes are upregulated under oxidative stress, facilitating enhanced substrate-selective degradation and proteostasis maintenance [21,30].

With respect to cytokine signaling, the IL-6-STAT3 axis has been implicated in the transcriptional regulation of autophagy-related genes; however, whether STAT3 directly targets CMA-specific loci-such as the HSPA8 promoter-remains experimentally unverified [145,146]. Likewise, IFN-γ can regulate autophagy and immune responses through the JAK-STAT1 signaling pathway [147], and can induce the expression of multiple STAT1-dependent genes in mesenchymal stem cell models [148]. Although CMA may be influenced by this pathway, direct experimental evidence linking IFN-γ to LAMP2A expression is still lacking.

Together, these findings suggest that CMA exhibits a high degree of plasticity in response to inflammatory stimuli, enabling adaptive fine-tuning of lysosomal proteostasis during immune activation. Nonetheless, the precise regulatory circuits and transcriptional mechanisms linking cytokine signaling to CMA activity remain to be elucidated through targeted molecular studies.

#### 4.2.2. The Potential Regulation of CMA by Toll-like Receptors (TLRs) Activation and Pathogen-Associated Molecular Patterns (PAMPs) Stimulation

Toll-like receptors (TLRs) act as central pattern-recognition receptors (PRRs) that detect pathogen-associated molecular patterns (PAMPs) and initiate innate immune signaling. Extensive studies have established that TLR engagement robustly modulates macroautophagy, thereby influencing pathogen clearance, antigen presentation, and inflammatory balance [149]. However, whether TLR signaling directly regulates CMA remains largely uncharacterized.

Preliminary observations and review analyses suggest that LAMP-2A expression in macrophages may undergo dynamic fluctuations upon stimulation with lipopolysaccharide (LPS) or synthetic PAMPs, implying that CMA might function as a stress-adaptive quality-control mechanism under these conditions [140]. Nonetheless, there is no experimental evidence to date showing that the canonical TLR downstream adaptors-MyD88 or TIR-domain-containing adaptor-inducing interferon-β (TRIF)-directly target or modify CMA core components such as LAMP-2A or Hsc70 [150].

At the functional level, TLR activation promotes dendritic cell (DC) maturation and enhances MHC II-dependent antigen presentation, thereby potentiating adaptive immune priming [151]. Moreover, loss of signaling adaptors DNAX-activating protein of 12 kDa (DAP12) or Fc receptor gamma chain (FcRγ) amplifies TLR-mediated responses, highlighting the intricate role of TLR pathways in DC regulation [152]. These processes are frequently accompanied by lysosomal remodeling and autophagy induction, yet the specific contribution of CMA remains speculative due to a lack of direct mechanistic validation.

Collectively, current evidence underscores a tight interplay between TLR/PAMP signaling and macroautophagy, whereas the involvement of CMA is supported only by scattered observations and indirect inference. Future studies integrating CMA-specific reporter systems (e.g., KFERQ-Dendra2) with immune cell-specific LAMP-2A knockout models will be essential to delineate the physiological relevance and mechanistic integration of CMA within TLR-driven immune responses.

#### 4.2.3. The Suppressive Role of Anti-Inflammatory Cytokines in CMA

Interleukin-10 (IL-10), a pivotal anti-inflammatory cytokine, plays a central role in the resolution phase of immune responses and the restoration of homeostasis. Previous studies have shown that IL-10 can inhibit starvation-induced macroautophagy in macrophages through the PI3K signaling pathway [153], acting in concert with metabolic reprogramming to promote the termination of inflammation [154,155]. These findings suggest that IL-10 may coordinate immune resolution via the dual modulation of autophagy and metabolism. However, direct evidence of IL-10-mediated regulation of CMA is currently lacking.

Temporally, IL-10 expression typically rises during the late stages of infection, coinciding with immune de-escalation and tissue recovery [156]. In parallel, CMA has been shown to exert cytoprotective effects under stress and inflammatory conditions by maintaining proteostasis [157], providing a theoretical rationale for potential functional interplay between IL-10 and CMA during the resolution phase of inflammation.

From a mechanistic standpoint, recent reviews have proposed that IL-10 may indirectly influence lysosomal activity through mTORC1-dependent signaling [158,159,160]. Given the central role of mTORC1 in autophagy and lysosomal regulation, it is plausible that IL-10 could attenuate CMA flux by modulating lysosomal stability or substrate trafficking. Nevertheless, there is no direct evidence that IL-10 specifically alters LAMP-2A or Hsc70 expression or affects the translocation efficiency of KFERQ-bearing substrates across the lysosomal membrane.

In summary, the relationship between IL-10 signaling and CMA activity remains largely hypothetical. Future investigations employing CMA-specific reporter systems (e.g., *KFERQ-Dendra2*) and LAMP-2A deficient models under conditions of IL-10 pathway activation will be essential to determine whether CMA contributes to immune resolution and the re-establishment of homeostatic equilibrium.

### 4.3. Dysregulation of CMA and Its Implications for Human Disease and Therapeutic Intervention

Beyond infectious diseases, CMA-related chaperone networks have also been discussed in a variety of situations including chronic inflammatory disorders; however, mechanistic and clinical evidence remains limited and is outside the scope of this infection-focused review.

#### 4.3.1. Pathogen-Mediated Interference with CMA and Mechanisms of Immune Evasion

Mounting evidence indicates that pathogens have evolved sophisticated strategies to subvert the host autophagy lysosome system, thereby facilitating immune evasion and intracellular persistence. In the case of hepatitis B virus (HBV), its X protein (HBx) has been shown to disrupt the localization of vacuolar-type H^+^-ATPase (V-ATPase), impairing lysosomal acidification and maturation, and consequently blocking autophagic substrate degradation [161]. Furthermore, Rab7-dependent fusion between autophagosomes and lysosomes is essential for HBV clearance, yet the virus suppresses Rab7 expression to avoid degradation and prolong intracellular survival [162,163]. Early studies revealed that HBV infection promotes the formation of autophagosomes without enhancing their subsequent degradative steps, resulting in an “incomplete autophagy” phenotype [163]. Nevertheless, no current evidence supports direct viral manipulation of core CMA components such as LAMP-2A or Hsc70, leaving open the question of whether HBV can exploit CMA to achieve immune evasion.

Intriguingly, recent research in RNA virus models has provided the first direct link between CMA activation and viral replication. The type 2 porcine reproductive and respiratory syndrome virus (PRRSV-2) was shown to upregulate ras-related protein Rab-18 (RAB18), thereby activating CMA and promoting degradation of lipid droplet associated proteins to remodel host metabolism in favor of viral replication [57]. This finding suggests that certain RNA viruses may manipulate not only macroautophagy but also CMA to reshape host metabolic and stress responses, expanding the known spectrum of autophagy-based immune evasion strategies.

In bacterial infections, *Staphylococcus aureus* has been reported to exploit autophagy to establish an intracellular niche conducive to its survival [164]. Its Agr quorum-sensing system inhibits autophagosome maturation, thereby shielding the bacterium from lysosomal degradation [165]. Similarly, *Mycobacterium tuberculosis* secretes virulence effectors such as SapM and ESAT-6, which compromise lysosomal integrity and suppress macroautophagic flux, enhancing bacterial persistence within host cells [166,167]. To date, however, no evidence has demonstrated direct bacterial interference with CMA-specific machinery.

Collectively, these findings underscore a broader pathogenic strategy in which microbes may not only inhibit macroautophagy but potentially target CMA as an unrecognized host defense pathway. Elucidating whether CMA flux alterations during infection represent a host stress response or an active pathogen-driven manipulation will be a crucial next step. Such insights could pave the way for host-directed therapeutic strategies centered on CMA activation to counteract pathogen-mediated immune evasion [76].

#### 4.3.2. Therapeutic Potential of CMA as a Regulatory Node in Immunometabolic Control

CMA functions as a pivotal node linking proteostasis, metabolic regulation, and immune homeostasis, and has been systematically recognized as a fundamental arm of the cellular quality-control and signaling network [168]. Notably, CMA activity declines progressively with age, a deterioration that compromises the clearance of misfolded or damaged proteins and is tightly associated with tissue homeostatic imbalance and immune senescence [76].

In multiple neurodegenerative disease models, pharmacological or genetic activation of CMA has been shown to alleviate protein-aggregation pathologies, including aberrant accumulation of Tau and α-synuclein [169]. Small-molecule studies further demonstrate that pharmacologic enhancement of LAMP-2A expression and CMA flux can restore intracellular proteostasis. For instance, all-trans retinoic acid (ATRA) derivatives have been shown in vitro to effectively activate CMA and promote substrate degradation [136,137], while the novel compound CA77.1 alleviates proteotoxic stress and preserves tissue integrity in models of retinal degeneration [170]. These findings collectively confirm the feasibility of pharmacological CMA modulation and highlight its therapeutic promise in protein aggregation-associated pathologies, particularly neurodegenerative diseases. Moreover, age-associated CMA decline is increasingly viewed as a driving factor of systemic functional decline, and its reactivation may delay degenerative aging phenotypes, reinforcing the concept of CMA as a viable target for anti-aging and neuroprotective interventions [171].

Although most evidence originates from neural systems, recent studies have extended the relevance of CMA to immune aging. In aged mice, restoration of LAMP-2A expression reestablishes CMA activity, leading to rebalanced T-cell subsets and preserved immune competence, thereby underscoring CMA’s role in maintaining immune equilibrium [172].

Beyond proteostasis, CMA also contributes to metabolic homeostasis through the selective degradation of metabolic regulators. Recent work revealed that CMA targets the mitochondrial E3 ubiquitin ligase membrane-associated ring-CH-type finger 5 (MARCHF5), preventing its aberrant accumulation and maintaining mitochondrial dynamics. When CMA function is intact, MARCHF5 turnover supports the balance of mitochondrial fission and fusion, thereby sustaining oxidative phosphorylation efficiency and cellular energy stability. In contrast, CMA deficiency results in excessive MARCHF5 accumulation, leading to mitochondrial fragmentation, reduced ATP generation, and metabolic collapse [173]. In parallel, nonimmune studies have identified direct crosstalk between CMA and energy-sensing pathways: under energy stress, AMPK phosphorylates the lipid droplet-associated protein PLIN2, promoting its Hsc70-mediated targeting to lysosomes via CMA and thus enabling dynamic lipid catabolism [174]. This mechanism not only places CMA under AMPK regulation, but also provides a conceptual framework for its potential role in immunometabolic adaptation.

Taken together, although pharmacological modulation of CMA remains in the preclinical stage and faces challenges such as compound specificity, delivery efficiency, and potential off-target effects, the pathway’s dual function in proteostasis and metabolic-immune integration positions it as a strategically valuable target. In contexts such as infectious disease, chronic inflammation, and immune dysfunction, CMA-targeted interventions may evolve into a promising host-directed therapeutic strategy. Future mechanistic and translational studies are warranted to define CMA’s regulatory circuitry and optimize its clinical applicability.

## 5. Clinical Relevance and Future Challenges of CMA in Infection

**Figure 4:**
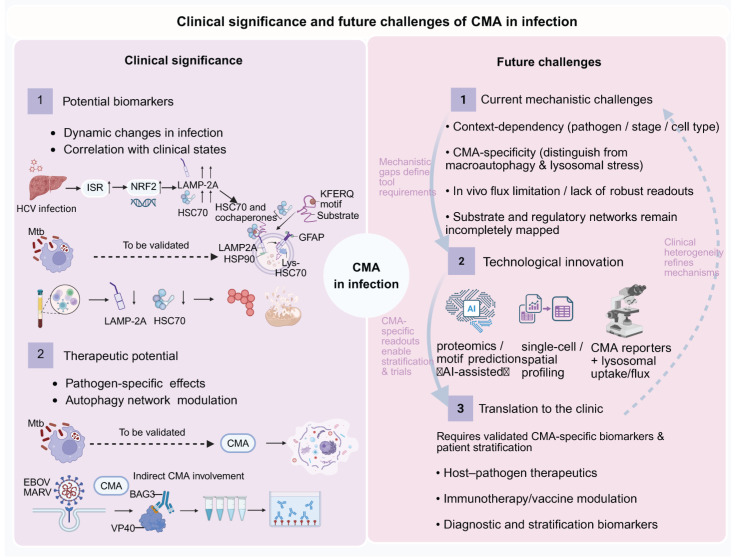
Clinical significance and future challenges of CMA in infection. This figure outlines the clinical significance and future challenges of CMA in infection, highlighting its potential for biomarkers and therapeutics, alongside unresolved mechanistic gaps. Technological advances and systems-level approaches will be essential to translate CMA modulation into viable diagnostic and host-directed therapeutic strategies. Created in BioRender. Li, C. (2026) https://BioRender.com/1nuzi3y (accessed on 15 January 2026).

CMA exhibits dynamic regulation during infection, with its core components-LAMP-2A and Hsc70-showing context-dependent alterations across various bacterial and viral models. These observations suggest that CMA actively participates in host–pathogen interactions and may possess both diagnostic and therapeutic potential, although supporting evidence remains largely exploratory. The clinical implications of CMA in infection, together with the major challenges and research priorities, are summarized in Figure 4.

### 5.1. Clinical Significance

#### 5.1.1. CMA-Associated Components as Biomarkers

Although alterations in LAMP-2A and Hsc70 have been reported across models, their steady-state abundance alone is unlikely to provide diagnostic specificity. This is because both proteins represent ubiquitous stress-responsive nodes that can be modulated by diverse conditions (e.g., malignancy, fasting, neurodegeneration, and systemic inflammation) [175]. Consistent with this limitation, conventional measurements of LAMP-2A/Hsc70 levels cannot reliably distinguish CMA from other lysosome-dependent degradation routes, and CMA activity is best assessed using flux-oriented readouts with LAMP-2A/Hsc70-dependence [176]. Therefore, rather than proposing LAMP-2A/Hsc70 as standalone diagnostic markers for infection, a more clinically plausible direction is to develop multi-parameter “CMA-related signatures” that integrate (i) CMA-proximal features (e.g., LAMP-2A/Hsc70 together with functional flux proxies where feasible), (ii) lysosomal stress markers, and (iii) pathogen- and tissue-contextual immune readouts. Such composite signatures could be evaluated primarily for patient stratification (e.g., hyperinflammatory vs. resolution phenotypes) or as pharmacodynamic markers in trials aiming to modulate the autophagy–lysosome network.

Despite the progress and some relevance of CMA’s potential for a diagnostic marker, it is critical to furhter investigate its significance. For instance, future cohort studies should explicitly control for major confounders that independently remodel proteostasis (age, metabolic state, cancer, and neurodegenerative comorbidities), and should validate whether any proposed CMA-related panel adds predictive value beyond established inflammatory biomarkers.

#### 5.1.2. Therapeutic Potential and Autophagy Network Modulation

CMA has been proposed as a potential host-directed therapeutic node in infectious diseases; however, current evidence remains largely preclinical and context-dependent, with outcomes varying by pathogen, cell type, and infection stage. In bacterial infection models (e.g., *Mycobacterium tuberculosis*), compounds like all-trans retinoic acid (ATRA) have been reported to enhance autophagy-lysosome activity and promote pathogen clearance; whether these effects reflect direct CMA modulation requires dedicated CMA-specific readouts [177].

In viral infections, components of the CMA-associated chaperone network may similarly participate in host defense. For instance, in Ebola virus (EBOV) and Marburg virus (MARV) infection models, the co-chaperone BCL2-associated athanogene 3 (BAG3) was found to bind the viral VP40 matrix protein, sequestering it away from budding sites and thereby restricting virion release [178]. While this mechanism does not directly implicate the canonical LAMP-2A-Hsc70-mediated CMA pathway, it suggests that CMA-associated proteostasis networks may influence viral protein handling and limit replication.

In emerging viral infections, disruption of the autophagy-lysosome axis has been increasingly recognized as a determinant of pathogenic injury. The SARS-CoV-2 ORF3a protein, for example, blocks autophagosome-lysosome fusion, leading to stalled autophagic flux, exacerbated inflammation, and tissue damage [179]. Several studies have demonstrated that small molecules such as spermidine and niclosamide can partially restore autophagic flux, which not only facilitates viral clearance but also mitigates excessive inflammatory responses [180]. Although these effects have primarily been attributed to macroautophagy, it remains plausible that CMA is concomitantly regulated under such stress conditions-a hypothesis that warrants investigation using CMA-specific reporter systems.

### 5.2. Current Challenges and Research Gaps

Despite rapid progress, research on CMA continues to face several major challenges.

#### 5.2.1. Lack of Specific In Vivo Monitoring Tools

Conventional assessments based on LAMP-2A or Hsc70 protein levels cannot reliably distinguish CMA from other lysosome-dependent degradation pathways, and real-time reporters capable of quantifying CMA flux under physiological conditions are still limited [21,33].

#### 5.2.2. Pathogen- and Cell-Type Heterogeneity

CMA regulation exhibits marked heterogeneity across different pathogens, cell types, and infection contexts. For example, bacterial infections often correlate with CMA activation that supports antigen processing and immune surveillance, whereas certain viruses appear to suppress CMA to facilitate persistent infection and immune evasion [33,131].

#### 5.2.3. Insufficient Knowledge of Nonimmune Cell Regulation

Studies of CMA in nonimmune cell populations-such as epithelial or barrier cells-remain scarce, leaving major gaps in understanding its spatiotemporal dynamics and tissue-specific regulatory logic [33]. Defining CMA with spatial and temporal resolution would directly address several mechanistic ambiguities in infection biology. Key questions that would benefit most from such analyses include: (i) which tissue compartments and cell types (immune vs. barrier/epithelial/stromal) exhibit the dominant CMA response at distinct stages of infection; (ii) whether CMA is preferentially engaged in pathogen-infected cells versus bystander cells exposed to inflammatory or metabolic stress; (iii) how CMA dynamics correlate with pathogen burden, cytokine gradients, and tissue-damage/repair programs over time; (iv) whether CMA activity is compartmentalized to specific lysosome subpopulations or subcellular regions that intersect with pathogen replication/trafficking niches; and (v) when CMA switches from protective proteostasis control to a pathway potentially exploited for immune evasion or persistence. Addressing these points will require time-resolved, tissue-resolved CMA flux reporters and integration with single-cell and spatial multi-omics in infection models [32,54,55,181,182].

#### 5.2.4. Unresolved Cross-System Integration

In addition, CMA likely interfaces with mitochondrial quality control, immunometabolic adaptation, and inflammasome activation, yet the causal interconnections among these systems remain poorly defined [183,184].

#### 5.2.5. Translational Limitations

Lack of standardized biomarkers, incomplete validation of CMA-targeting drugs, and concerns over systemic activation hinder clinical application [21,33,185].

### 5.3. Technological and Translational Innovations

Addressing these challenges will require multidisciplinary integration spanning cell biology, bioengineering, and computational modeling.

The development of real-time CMA reporter systems-such as KFERQ-Gamillus-offers new avenues for visualizing CMA dynamics in living cells and tissues [32,54,55]. Complementarily, single-cell multi-omics approaches promise to unravel cell-type-specific heterogeneity and the cross-talk between immune and nonimmune compartments [181,182].

In vivo, humanized mouse models and complex infection systems provide valuable platforms to interrogate CMA function under physiologically relevant conditions [186,187]. Meanwhile, emerging synthetic biology and systems biology frameworks may enable quantitative modeling of CMA signaling networks, helping identify regulatory bottlenecks and potential drug targets [188,189,190].

At the translational interface, while CMA-specific nanodelivery systems have yet to be realized, advances in macroautophagy-targeted therapeutics offer promising design principles for enhancing specificity, tissue selectivity, and pharmacological safety [191,192].

Taken together, the next frontier of CMA research lies in bridging mechanistic insight with technological innovation to enable precision modulation of CMA in infection, inflammation, and immune aging-paving the way toward clinically viable host-directed therapies.

### 5.4. Translational Outlook

The future trajectory of CMA research is likely to unfold along three major axes:

First, host–pathogen interaction as a therapeutic frontier. Several pathogens-including hepatitis C virus (HCV) and porcine reproductive and respiratory syndrome virus (PRRSV)-have been shown to exploit the Hsc70-LAMP-2A axis to remodel host proteostasis, providing mechanistic justification for CMA modulation as a potential intervention point to restrict infection [193,194].

Second, immunotherapeutic potential. Recent findings about CMA in T-cell activation and functional persistence suggest that targeted enhancement of CMA may augment vaccine efficacy, fine-tune immune tolerance, or mitigate hyperinflammatory responses, positioning CMA as an immunomodulatory lever in both infection and inflammatory disease contexts [52].

Third, molecular stratification and diagnostic application. Alterations in LAMP-2A/Hsc70 expression have been correlated with disease progression and adverse prognosis in HCV infection, cirrhosis-associated hepatocellular carcinoma, and lung cancer, underscoring the potential of CMA components as biomarkers for clinical stratification [195,196,197]. Furthermore, the emerging roles of CMA in antigen processing and immune tolerance may inform novel strategies for autoimmune and cancer immunotherapy [198,199,200].

Collectively, although CMA research in infectious settings remains at an early stage, it is increasingly recognized as a central integrator of proteostasis, metabolic regulation, and immune function. With the aid of next-generation analytical platforms and systems-level infection models, future work will likely elucidate the mechanistic underpinnings of CMA in host defense and accelerate its translation into diagnostic and therapeutic innovations for infectious and immune-mediated diseases. In summary, this section outlines a roadmap from exploratory clinical associations to mechanism-informed and technology-enabled translation, as summarized in Figure 4.

## Figures and Tables

**Table 1:** CMA-linked mechanisms supported by CMA-specific or component-level evidence in infection contexts (Tier 1–3).

Pathogen (Type)	CMA-Related Evidence (Summary)	Evidence Tier	CMA Flux Readout?	LAMP-2A/Hsc70 Dependency (Genetic ± Rescue)?	Net effect on Infection Outcome	Key Pathways/Nodes Implicated	Representative Models/Readouts	Key Citations
SARS-CoV-2 (RNA virus)	Component-level association. LAMP-2A was implicated in viral 5′/3′ UTR RNP complexes; LAMP-2A overexpression reduced viral RNA abundance. CMA flux and CMA-specific causality were not established, therefore interpreted as LAMP-2A-associated rather than CMA-dependent.	Tier 3	No	No	Anti-viral (suggested)	UTR RNP–LAMP-2A association; CMA-related components	Viral RNA abundance; LAMP-2A perturbation/overexpression; infection/replication readouts	[56]
PRRSV-2 (RNA virus)	CMA-specific causal mechanism. PRRSV-2 upregulates RAB18 and hijacks CMA-mediated lipolysis to support replication. The study provides CMA flux evidence and demonstrates LAMP-2A/Hsc70 dependency with genetic perturbation and rescue/validation, establishing CMA dependence beyond general autophagy–lysosome effects.	Tier 1	Yes	Yes	Pro-viral (supported)	RAB18 → CMA-mediated lipolysis; lipid droplet remodeling	Infection in relevant host cells; CMA flux reporter/uptake readouts; LAMP-2A/Hsc70 loss-of-function ± rescue/validation; viral replication/viral RNA/protein readouts; lipid droplet–associated protein turnover	[57]

Abbreviations: CMA, chaperone-mediated autophagy; RNP, ribonucleoprotein; UTR, untranslated region.

## Data Availability

No new data were created or analyzed in this study. Data sharing is not applicable to this article.

## Supplementary Materials

The following supporting information can be downloaded at: https://www.mdpi.com/article/10.3390/ijms27031164/s1.
