# Supplementary material for: Critical Roles and Molecular Mechanisms of Chaperone-Mediated Autophagy in Infections
Source: Int J Mol Sci. 2026 Jan 23;27(3):1164. doi: 10.3390/ijms27031164 (PMC12897289; doi:10.3390/ijms27031164)
Supplement: Supplementary file 1 [file ijms-27-01164-s001.zip › Table S2.pdf]

Pathogen / context	Hypothesis-level CMA link (suggested, not proven)	What is missing for CMA-specific assignment	Key pathways / nodes implicated	Key citations
PRR stimulation (LPS; microglial activation model)	Deficient CMA is associated with amplified inflammatory signaling (e.g., p300–NF-κB–NLRP3), supporting a conceptual role for CMA in restraining excessive inflammation.	Infection-context CMA flux mapping; pathogen-driven CMA activation; dependency/rescue in infection models	p300–NF-κB–NLRP3; inflammatory cytokines	[120]
Fungal infections (e.g., Candida / Cryptococcus; stress contexts)	Fungal infection–associated oxidative/ER stress could intersect with CMA regulation; ER stress can couple to CMA via LAMP-2A regulation in non-infectious settings.	Direct fungus→CMA flux evidence in infection; LAMP-2A/Hsc70 dependency/rescue	Oxidative stress; ER stress/UPR; p38–LAMP-2A coupling	[101]
Plasmodium liver stage (parasite)	Parasite-induced ER stress/UPR in hepatocytes could potentially interface with CMA regulation.	CMA flux reporter/uptake assays; LAMP-2A/Hsc70 dependency/rescue in infection context	UPR/ER stress programs	[22,128]
Toxoplasma gondii (parasite)	Parasite-driven inflammatory and stress pathways (NF-κB/p38; ISR/eIF2α–ATF4) may remodel proteostasis/autophagy programs with possible indirect relevance to CMA.	CMA substrate clearance/flux readouts; LAMP-2A/Hsc70 dependency/rescue; separation from macroautophagy effects	NF-κB (GRA15); p38 (GRA24); ISR/eIF2α–ATF4	[132–135]
