# Supplementary material for: Critical Roles and Molecular Mechanisms of Chaperone-Mediated Autophagy in Infections
Source: Int J Mol Sci. 2026 Jan 23;27(3):1164. doi: 10.3390/ijms27031164 (PMC12897289; doi:10.3390/ijms27031164)
Supplement: Supplementary file 1 [file ijms-27-01164-s001.zip › Table S1.pdf]

Pathogen (type)	Why excluded (not CMA-specific)	Dominant pathway affected	Representative models / readouts	Key citations
HBV (DNA virus)	HBx disrupts lysosomal acidification/maturation and impairs lysosomal degradation; Rab7-dependent fusion contributes to viral clearance. No evidence for direct manipulation of LAMP-2A/Hsc70 or CMA flux.	Lysosomal maturation/acidification; autophagosome–lysosome fusion	Viral replication; lysosome maturation markers; autophagy–lysosome fusion readouts	[161–163]
Staphylococcus aureus (bacterium)	Exploits autophagy for intracellular survival and modulates autophagosome maturation (e.g., via Agr system). No evidence for CMA machinery involvement (LAMP-2A/Hsc70).	Macroautophagy / autophagosome maturation	Intracellular burden; autophagy flux markers	[164,165]
Mycobacterium tuberculosis (bacterium)	Virulence effectors compromise lysosomal integrity and suppress macroautophagic flux; CMA-specific interference not shown.	Lysosomal integrity; macroautophagy flux; mTOR-related modulation	Intracellular burden; autophagy flux; lysosome integrity readouts	[166,167]
